# Dados de Vida Real sobre o Uso da Hidroxicloroquina ou da Cloroquina Combinadas ou Não à Azitromicina em Pacientes com Covid-19: Uma Análise Retrospectiva no Brasil

**DOI:** 10.36660/abc.20220935

**Published:** 2023-10-03

**Authors:** Maíra Viana Rego Souza-Silva, Daniella Nunes Pereira, Magda Carvalho Pires, Isabela Muzzi Vasconcelos, Alexandre Vargas Schwarzbold, Diego Henrique de Vasconcelos, Elayne Crestani Pereira, Euler Roberto Fernandes Manenti, Felício Roberto Costa, Filipe Carrilho de Aguiar, Fernando Anschau, Frederico Bartolazzi, Guilherme Fagundes Nascimento, Heloisa Reniers Vianna, Joanna d’Arc Lyra Batista, Juliana Machado-Rugolo, Karen Brasil Ruschel, Maria Angélica Pires Ferreira, Leonardo Seixas de Oliveira, Luanna Silva Monteiro Menezes, Patricia Klarmann Ziegelmann, Marcela Gonçalves Trindade Tofani, Maria Aparecida Camargos Bicalho, Matheus Carvalho Alves Nogueira, Milton Henriques Guimarães-Júnior, Rúbia Laura Oliveira Aguiar, Danyelle Romana Alves Rios, Carisi Anne Polanczyk, Milena Soriano Marcolino

**Affiliations:** 1 Faculdade de Medicina Universidade Federal de Minas Gerais Belo Horizonte MG Brasil Faculdade de Medicina – Universidade Federal de Minas Gerais, Belo Horizonte, MG – Brasil; 2 Departamento de Estatística Instituto de Ciências Exatas Universidade Federal de Minas Gerais Belo Horizonte MG Brasil Departamento de Estatística – Instituto de Ciências Exatas (ICEx) – Universidade Federal de Minas Gerais, Belo Horizonte, MG – Brasil; 3 Hospital Universitário de Santa Maria Universidade Federal de Santa Maria Santa Maria RS Brasil Hospital Universitário de Santa Maria (EBSERH) – Universidade Federal de Santa Maria, Santa Maria, RS – Brasil; 4 Hospital SOS Cárdio Florianópolis SC Brasil Hospital SOS Cárdio, Florianópolis, SC – Brasil; 5 Instituto de Medicina Vascular Hospital Mãe de Deus Porto Alegre RS Brasil Instituto de Medicina Vascular – Hospital Mãe de Deus, Porto Alegre, RS – Brasil; 6 Hospital Municipal Odilon Behrens Belo Horizonte MG Brasil Hospital Municipal Odilon Behrens, Belo Horizonte, MG – Brasil; 7 Hospital das Clínicas Universidade Federal de Pernambuco Recife PE Brasil Hospital das Clínicas da Universidade Federal de Pernambuco, Recife, PE – Brasil; 8 Programa de Avaliação e Produção de Tecnologias para o Sistema Único de Saúde Hospital Nossa Senhora da Conceição Hospital Cristo Redentor Porto Alegre RS Brasil Programa de Avaliação e Produção de Tecnologias para o Sistema Único de Saúde - Hospital Nossa Senhora da Conceição e Hospital Cristo Redentor, Porto Alegre, RS – Brasil; 9 Hospital Santo Antônio Curvelo MG Brasil Hospital Santo Antônio, Curvelo, MG – Brasil; 10 Hospital Unimed Belo Horizonte MG Brasil Hospital Unimed, Belo Horizonte, MG – Brasil; 11 Hospital Universitário Ciências Médicas Belo Horizonte MG Brasil Hospital Universitário Ciências Médicas, Belo Horizonte, MG – Brasil; 12 Hospital Mãe de Deus Universidade Federal da Fronteira Sul Chapecó SC Brasil Hospital Mãe de Deus – Universidade Federal da Fronteira Sul, Chapecó, SC – Brasil; 13 IATS CNPq Porto Alegre RS Brasil Instituto de Avaliação de Tecnologia em Saúde (IATS/CNPq), Porto Alegre, RS – Brasil; 14 Hospital das Clínicas Faculdade de Medicina de Botucatu Botucatu SP Brasil Hospital das Clínicas da Faculdade de Medicina de Botucatu, Botucatu, SP – Brasil; 15 Hospital Mãe de Deus Hospital Universitário de Canoas Universidade Federal do Rio Grande do Sul Porto Alegre RS Brasil Hospital Mãe de Deus – Hospital Universitário de Canoas - Universidade Federal do Rio Grande do Sul, Porto Alegre, RS – Brasil; 16 IATS CNPQ Porto Alegre RS Brasil Instituto de Avaliação de Tecnologia em Saúde (IATS/CNPQ), Porto Alegre, RS – Brasil; 17 Hospital de Clínicas de Porto Alegre Porto Alegre RS Brasil Hospital de Clínicas de Porto Alegre, Porto Alegre, RS – Brasil; 18 Hospital Santa Rosália Teófilo Otoni MG Brasil Hospital Santa Rosália, Teófilo Otoni, MG – Brasil; 19 Hospital Luxemburgo Belo Horizonte MG Brasil Hospital Luxemburgo, Belo Horizonte, MG – Brasil; 20 Hospital Tacchini Bento Gonçalves RS Brasil Hospital Tacchini, Bento Gonçalves, RS – Brasil; 21 Hospital Semper Belo Horizonte MG Brasil Hospital Semper, Belo Horizonte, MG – Brasil; 22 Departamento de Medicina Interna Faculdade de Medicina Hospitais Fundação Hospitalar do Estado de Minas Gerais Belo Horizonte MG Brasil Departamento de Medicina Interna, Faculdade de Medicina - Hospitais Fundação Hospitalar do Estado de Minas Gerais (FHEMIG), Belo Horizonte, MG – Brasil; 23 Hospitais da Rede Mater Dei Belo Horizonte MG Brasil Hospitais da Rede Mater Dei, Belo Horizonte, MG – Brasil; 24 Hospital Márcio Cunha Ipatinga MG Brasil Hospital Márcio Cunha, Ipatinga, MG – Brasil; 25 Hospital Metropolitano Doutor Célio de Castro Belo Horizonte MG Brasil Hospital Metropolitano Doutor Célio de Castro, Belo Horizonte, MG – Brasil; 26 Universidade Federal de São João del-Rei Divinópolis MG Brasil Universidade Federal de São João del-Rei, Divinópolis, MG – Brasil; 27 Universidade Federal do Rio Grande do Sul Porto Alegre RS Brasil Universidade Federal do Rio Grande do Sul, Porto Alegre, RS – Brasil; 28 Instituto de Avaliação de Tecnologias em Saúde CNPq Porto Alegre RS Brasil Instituto de Avaliação de Tecnologias em Saúde (IATS/CNPq), Porto Alegre, RS – Brasil; 29 Hospital Moinhos de Vento Porto Alegre RS Brasil Hospital Moinhos de Vento, Porto Alegre, RS – Brasil; 30 Departamento de Clínica Médica Faculdade de Medicina Universidade Federal de Minas Gerais Belo Horizonte MG Brasil Departamento de Clínica Médica – Faculdade de Medicina – Universidade Federal de Minas Gerais, Belo Horizonte, MG – Brasil; 31 Centro de Telessaúde Hospital Universitário Universidade Federal de Minas Gerais Belo Horizonte MG Brasil Centro de Telessaúde do Hospital Universitário da Universidade Federal de Minas Gerais, Belo Horizonte, MG – Brasil

**Keywords:** Cloroquina, Hidroxicloroquina, azitromicina, covid-19

## Abstract

**Fundamento:**

Apesar da ausência de evidência mostrando benefícios da hidroxicloroquina e da cloroquina combinadas ou não à azitromicina no tratamento da covid-19, esses medicamentos têm sido amplamente prescritos no Brasil.

**Objetivos:**

Avaliar desfechos, incluindo moralidade hospitalar, alterações eletrocardiográficas, tempo de internação, admissão na unidade de terapia intensiva, e necessidade de diálise e de ventilação mecânica em pacientes hospitalizados com covid-19 que receberam cloroquina ou hidroxicloroquina, e comparar os desfechos entre aqueles pacientes e seus controles pareados.

**Métodos:**

Estudo multicêntrico retrospectivo do tipo coorte que incluiu pacientes com diagnóstico laboratorial de covid-19 de 37 hospitais no Brasil de março a setembro de 2020. Escore de propensão foi usado para selecionar controles pareados quanto a idade, sexo, comorbidades cardiovasculares, e uso de corticosteroides durante a internação. Um valor de p<0,05 foi considerado estatisticamente significativo.

**Resultados:**

Dos 7850 pacientes com covid-19, 673 (8,6%) receberam hidroxicloroquina e 67 (0,9%) cloroquina. A idade mediana no grupo de estudo foi 60 (46-71) anos e 59,1% eram mulheres. Durante a internação, 3,2% dos pacientes apresentaram efeitos adversos e 2,2% necessitaram de interromper o tratamento. Alterações eletrocardiográficas foram mais prevalentes no grupo hidroxicloroquina/cloroquina (13,2% vs. 8,2%, p=0,01), e o prolongamento do intervalo QT corrigido foi a principal diferença (3,6% vs. 0,4%, p<0,001). O tempo mediano de internação hospitalar foi maior no grupo usando CQ/HCQ em relação aos controles (9,0 [5,0-18,0] vs. 8,0 [4,0-14,0] dias). Não houve diferenças estatisticamente significativas entre os grupos quanto a admissão na unidade de terapia intensiva (35,1% vs. 32,0%; p=0,282), ventilação mecânica invasiva (27,0% vs. 22,3%; p=0,074) ou mortalidade (18,9% vs. 18,0%; p=0,682).

**Conclusão:**

Pacientes com covid-19 tratados com cloroquina ou hidroxicloroquina apresentaram maior tempo de internação hospitalar, em comparação aos controles. Não houve diferença em relação a admissão em unidade de terapia intensiva, necessidade de ventilação mecânica e mortalidade hospitalar.

## Figura Central

**Figure:**
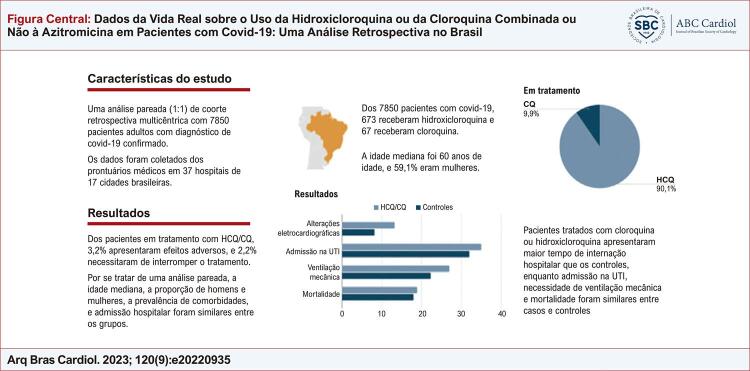
: Dados da Vida Real sobre o Uso da Hidroxicloroquina ou da Cloroquina Combinada ou Não à Azitromicina em Pacientes com Covid-19: Uma Análise Retrospectiva no Brasil

## Introdução

A pandemia da covid-19 provocou um esforço global sem precedentes na busca de tratamentos efetivos no combate à doença. Nesse contexto, a hidroxicloroquina (HCQ) e a cloroquina (CQ) chamaram a atenção da comunidade científica devido à evidência, *in vitro*, de atividade antiviral e efeito imunomodulatório, o que, em teoria, preveniria a tempestade de citocina.^1^ Estudos *in vitro* também mostraram que a azitromicina (AZT) poderia ter um efeito sinérgico sobre os efeitos da HCQ/CQ contra o SARS-CoV-2.^2^ Além disso, ambas as medicações são acessíveis e prontamente disponíveis em todo o mundo, possuem um perfil de segurança bem conhecido, e rapidamente se tornaram tratamentos potenciais da covid-19.^2^

Com base nessa informação preliminar encorajadora, agências reguladoras importantes, tais como a *Food and Drug Administration* (FDA) e a *European Medicines Agency* (EMA), autorizaram a utilização da HCQ e da CQ fora de ensaios clínicos em março de 2020, dada a situação emergencial.^3,4^

Atualmente, mesmo com resultados robustos de grandes ensaios clínicos randomizados (ECRs), como o RECOERY^5^ e o SOLIDARITY-WHO,^6^ e várias metanálises mostrando que não há evidência de benefício dessa terapia, e sim até evidência de prejuízo,^7,8^ alguns médicos e diretrizes brasileiras continuaram a recomendar o uso dessa medicação no tratamento da covid-19.^9^ Assim, este estudo teve como objetivo avaliar desfechos clínicos e eletrocardiográficos de pacientes internados com covid-19, que receberam HCQ ou CQ, com ou sem AZT, de uma grande coorte de hospitais brasileiros, e comparar os desfechos entre esses pacientes e controles pareados.

## Métodos

### Delineamento do estudo

O presente estudo é um subestudo do Registro covid-19 Brasileiro, um estudo de coorte multicêntrica, que incluiu pacientes consecutivos com diagnóstico de covid-19 confirmado laboratorialmente.^10,11^ Este estudo segue as diretrizes STROBE (*Strengthening the Reporting of Observational Studies in Epidemiology*).^12^ A Comissão Nacional de Ética em Pesquisa aprovou o desenvolvimento do estudo e dispensou a necessidade de consentimento individual, devido às circunstância da pandemia e análise baseada somente em dados de pacientes não identificados.

A presente análise incluiu pacientes admitidos em 37 hospitais participantes, de 17 cidades brasileiras, de março a setembro de 2020.^10,11^ Não houve perdas devido à natureza retrospectiva do estudo. Pacientes com valores faltantes nas diferentes variáveis não foram excluídos, e não houve valores faltantes relacionados à exposição. Optamos por não aplicar nenhum outro critério de exclusão, uma vez que nosso objetivo foi elaborar uma análise observacional da vida real.

### Coleta de dados

Pesquisadores treinados coletaram os dados dos pacientes dos prontuários médicos utilizando o sistema de captura de dados eletrônicos REDCap® (*Research Data Capture*) disponibilizado pelo Centro de Telessaúde do Hospital das Clínicas da Universidade Federal de Minas Gerais.^13,14^ Os dados incluíram características demográficas e clínicas, avaliação clínica na internação, dados laboratoriais, de imagem e eletrocardiográficos, intervenções terapêuticas, e desfechos.^10^ Para assegurar confiabilidade e monitorar a qualidade dos dados, todas as informações foram submetidas à uma verificação automática periódica para identificar valores discrepantes (*outliers*) e possíveis inconsistências.

### Desfechos

O desfecho primário avaliado foi mortalidade hospitalar por covid-19. Desfechos secundários incluíram anormalidades eletrocardiográficas (ritmo, frequência cardíaca, intervalo QT corrigido, anormalidades estruturais, bloqueios de ramo e taquicardias), tempo de internação hospitalar, admissão na Unidade de Terapia Intensiva (UTI) e necessidade de diálise e de ventilação mecânica invasiva.

### Análise estatística

Para a análise descritiva, as características clínicas e demográficas dos pacientes foram representadas por distribuição de frequência, usando mediana e intervalo interquartil para variáveis contínuas, uma vez que não apresentaram distribuição normal, e números e porcentagens para as contagens. Pacientes que receberam HQC ou CQ com ou sem AZT (HCQ/CQ + AZT) em qualquer dosagem foram comparados a controles pareados (pacientes que não receberam esse tratamento) usando o teste do qui-quadrado e o teste exato de Fisher para variáveis categóricas e o teste de soma dos postos de Wilcoxon para variáveis contínuas. No segundo caso, o teste de Kolmogorov-Smirnov foi aplicado para verificar a normalidade dos dados.

Para fins de comparação, um modelo de escores de propensão (incluindo idade, sexo, número de comorbidades cardiovasculares, hospital de origem e uso de corticoides) foi estimado por regressão logística para ajuste de potenciais variáveis de confusão e pareamento dos pacientes com controles. O grupo controle foi definido pelo escore de propensão mais próximo aos pacientes tratados com CQ (até 0,17 desvio padrão do logit do escore de propensão, em uma escala de 0-1,00), usando o pacote MatchIt no software R.

Para os desfechos admissão na UTI, necessidade de diálise e de ventilação mecânica, os pacientes que usaram HCQ/CQ + AZT após sua ocorrência foram excluídos.

Os resultados foram considerados estatisticamente significativos em um nível de significância de 5%. A análise estatística foi realizada com o software R para estatística computacional (versão 4.2).

## Resultados

Dos 7850 pacientes internados com diagnóstico confirmado de covid-19, 725 (9,2%) receberam HCQ/CQ com ou sem AZT para o tratamento da covid-19 durante a internação, 659 (90,9%) foram tratados com HCQ, 67 (9,2) com CQ e 640 (88,3%) receberam AZT simultaneamente (Tabela Suplementar 1). Desses, 673 pacientes que receberam HCQ/CQ conseguiram ser pareados e a acurácia do modelo de propensão foi 0,91. As diferenças médias padronizadas das principais covariáveis antes e após o pareamento são apresentadas na Tabela Suplementar 2.

A prevalência de comorbidades foi similar entre os dois grupos, como apresentado na Tabela 1, exceto por doença renal crônica, que foi mais frequente em controles. Quanto às características clínicas à admissão, o grupo HCQ/CQ + AZT apresentou uma leve redução na relação SpO_2_/FiO_2_ em comparação aos controles. As características dos pacientes que foram avaliados quanto à ventilação mecânica invasiva, diálise e admissão em UTI estão descritas na Tabela Suplementar 3.

**Tabela 1:** – Características demográficas e clínicas dos pacientes com covid-19 em tratamento com hidroxicloroquina (HCQ) e a cloroquina (CQ) e controles pareados*

Características	HCQ/CQ + com ou sem AZT N= 673^1^	Controles N= 673^1^	Valor p^2^
**Idade (anos)**	58,0 (46, 70)	60,0 (46, 71)	0,186
**Sexo feminino**	376 (55,9)	398 (59,1)	0,247
**Doenças cardiovasculares**			
Hipertensão	335 (49,8)	351 (52,2)	0,413
Insuficiência cardíaca crônica	37 (5,5)	33 (4,9)	0,713
Doença arterial coronariana	26 (3,9)	23 (3,4)	0,771
Acidente vascular cerebral isquêmico	18 (2,7)	20 (3,0)	0,869
Fibrilação ou *flutter* atrial	17 (2,5)	14 (2,1)	0,716
**Doenças respiratórias**			
Asma	53 (7,9)	38 (5,6)	0,129
DPOC	45 (6,7)	33 (4,9)	0,199
**Doenças metabólicas**			
Diabetes mellitus	196 (29,1)	179 (26,6)	0,331
Obesidade	133 (19,8)	113 (16,8)	0,180
**Outras condições**			
Doença psiquiátrica	48 (7,1)	44 (6,5)	0,746
Câncer ativo	40 (5,9)	57 (8,5)	0,911
Doença renal crônica	30 (4,5)	49 (7,3)	0,037
Doença reumatológica	21 (3,1)	14 (2,1)	0,304
Cirrose	3 (0,4)	5 (0,7)	0,726
**Características clínicas na apresentação**			
Ventilação mecânica invasiva	52 (7,3)	46 (6,5)	0,591
SpO_2_/FiO_2_	438,1 (317,0, 457,1)	447,6 (335,7, 461,9)	0,011
**Hábitos de estilo de vida**			
Alcoolismo	15 (2,2)	25 (3,7)	0,149
Tabagismo	85 (12,6)	87 (12,9)	0,935
**Uso de medicamentos durante a internação**			
Antibióticos (exceto azitromicina)	550 (81,7)	522 (77,6)	
Anticoagulantes	596 (88,6)	578 (85,9)	
Corticosteroides	375 (55,7)	356 (52,9)	

* Pareados por idade, sexo, número de comorbidades cardiovasculares, hospital de origem, e uso de corticosteroide. ^1^n (%); Mediana (intervalo interquartil) ^2^teste do qui-quadrado de Pearson, teste de soma dos postos de Wilcoxon, teste exato de Fisher; AZT: azitromicina; DPOC: doença pulmonar obstrutiva crônica; SpO_2_: saturação periférica de oxigênio; FiO_2_: fração de oxigênio inspirado.

Em relação à posologia (Tabela 2), a maioria dos pacientes usou HCQ (90,1%) com ou sem AZT, na dose de 850mg no primeiro dia, seguido de 450 mg diariamente durante o tratamento. O tempo mediano de tratamento foi de cinco dias. Trinta pacientes apresentaram efeitos colaterais que foram atribuídos à medicação pela equipe médica assistente, com prolongamento do intervalo QT e sintomas gastrointestinais como os mais comuns. A terapia foi suspensa em 15 pacientes devido aos efeitos adversos.

**Tabela 2:** – Posologia da cloroquina e da hidroxicloroquina e efeitos colaterais nos pacientes com covid-19 (n=673)

Medicamento usado	n (%)
Cloroquina	65 (9,9)
Hidroxicloroquina	612 (90,1)
Com azitromicina	598 (88,1)
**Posologia da cloroquina**	
450 mg a cada 12 horas no primeiro dia + 450 mg a cada 24 horas nos dias seguintes	45 (67,2)
500 mg a cada 12 horas no primeiro dia + 250 mg a cada 12 horas nos dias seguintes	7 (10,4)
Outros	15 (23,4)
Duração da terapia (dias)	5,0 (3,0 - 6,0)
**Posologia da hidroxicloroquina**	
400 mg a cada 12 horas no primeiro dia + @@200 mg a cada 12 horas nos dias seguintes	252 (59,6)
400 mg a cada 24 horas	149 (35,2)
400 mg a cada 12 horas no primeiro dia + 200 mg a cada 8 horas nos dias seguintes	21 (5,0)
200 mg a cada 8 horas	1 (0,2)
Outros	195 (46,1)
Duração da terapia (dias)	5,0 (4,0 - 6,0)
Efeitos colaterais da medicação	30 (4,4)
Taquicardia ventricular monomórfica	1 (0,1)
Prolongamento do intervalo QT	16 (2,4)
Sintomas gastrointestinais	5 (0,7)
Fibrilação/*flutter* atrial	2 (0,3)
Disfunção cardíaca não isquêmica	1 (0,1)
Outros efeitos adversos	8 (1,2)
Suspensão da terapia por efeitos adversos	15 (2,2)

Menos da metade dos pacientes tratados apresentou registro de eletrocardiograma (ECG) nas primeiras 24 horas de admissão. A maioria dos pacientes estavam em ritmo sinusal e alterações primária de repolarização foram o achado eletrocardiográfico mais comum.

Para os desfechos de ECG (Tabela 3), os pacientes do grupo tratado apresentaram maior frequência de alterações eletrocardiográfica durante a internação. Prolongamento do intervalo QT foi a alteração mais comum, e sua frequência foi maior nos pacientes tratados com HCQ/CQ em comparação aos controles. Taquicardia supraventricular foi mais frequente nos controles. Não foram observadas outras diferenças estatisticamente significativas entre os grupos.

**Tabela 3:** – Achados eletrocardiográficos na admissão e desfechos durante a internação hospitalar dos pacientes com covid-19 tratados com hidroxicloroquina (HCQ) ou cloroquina (CQ) com ou sem azitromicina (AZT) e indivíduos controles

Características	HCQ/CQ + com ou sem AZT N = 673^1^	Controles N = 673^1^	Valor p^2^
Eletrocardiograma na admissão	285 (42,3)	182 (27,0)	<0,001
Frequência cardíaca, bpm	83,0 (74,0, 91,0)	85,0 (74,0, 96,0)	0,063
Intervalo QT, ms	360,0 (320,0, 406,0)	360,0 (320,0, 400,0)	0,060
Ritmo sinusal	262 (91,9)	155 (85,2)	0,016
Alterações de repolarização primária	78 (27,6)	50 (27,5)	>0,999
Bloqueio de ramo direito	13 (4,6)	13 (7,1)	0,337
Fibrilação/*flutter* atrial	7 (2,5)	9 (4,9)	0,249
Hemibloqueio anterior esquerdo	6 (2,1)	6 (3,3)	0,630
Bloqueio de ramo esquerdo	4 (1,4)	3 (1,6)	>0,999
Hipertrofia ventricular esquerda com alterações de ST-T	4 (1,4)	3 (1,6)	>0,999
Ritmo de marca-passo	3 (1,1)	3 (1,6)	0,683
Bloqueio AV de primeiro grau	2 (0,7)	3 (1,6)	0,384
Ondas Q patológicas	1 (0,4)	0 (0,0)	>0,999
Taquicardia ventricular monomórfica	0 (0,0)	1 (0,5)	0,153
Taquicardia supraventricular	0 (0,0)	1 (0,5)	0,153
Bloqueio atrioventricular total	0 (0,0)	2 (1,1)	0,153
Desfechos eletrocardiográficos	89 (13,2)	55 (8,2)	0,004
Fibrilação/*flutter* atrial	24 (3,6)	20 (3,0)	0,646
Prolongamento do intervalo QT	26 (3,6)	3 (0,4)	<0,001
Alterações primárias da repolarização	16 (2,4)	12 (1,8)	0,567
Hipertrofia ventricular esquerda com alterações de ST-T	5 (0,7)	5 (0,7)	>0,999
Bloqueio atrioventricular de primeiro grau	5 (0,7)	4 (0,6)	>0,999
Bloqueio de ramo direito	5 (0,7)	2 (0,3)	0,452
Extrassístoles ventriculares	2 (0,3)	5 (0,7)	0,452
Ritmo de marca-passo	2 (0,3)	2 (0,3)	>0,999
Taquicardia ventricular polimórfica	2 (0,3)	2 (0,3)	>0,999
Bloqueio de ramo esquerdo	2 (0,3)	2 (0,3)	>0,999
Hemibloqueio anterior esquerdo	1 (0,1)	1 (0,1)	>0,999
Extrassístoles supraventriculares	1 (0,1)	1 (0,1)	>0,999
Bloqueio atrioventricular de segundo grau	1 (0,1)	0 (0,0)	>0,999
Taquicardia supraventricular	0 (0,0)	6 (0,9)	0,031

Pareados por idade, sexo, número de comorbidades cardiovasculares, hospital de origem, e uso de corticosteroide. ^1^n (%); Mediana (intervalo interquartil). ^2^teste do qui-quadrado de Pearson, teste de soma dos postos de Wilcoxon, teste exato de Fisher.

Em relação aos desfechos (Tabela 4), o tempo mediano de internação hospitalar foi mais longo no grupo HCQ/CQ + AZT em relação aos controles. Não houve diferença estatisticamente significativa na necessidade de internação em UTI, ventilação mecânica ou diálise e mortalidade hospitalar entre os grupos.

**Tabela 4:** – Comparação dos desfechos clínicos de pacientes com covid-19 tratados com hidroxicloroquina (HCQ) ou cloroquina (CQ) com ou sem azitromicina (AZT) e indivíduos controles*

Características	HCQ/CQ + com ou sem AZT N = 673^1^	Casos válidos n (%)	Controles N = 673^1^	Casos válidos n (%)	Valor p^2^
Tempo de internação (dias)	9,0 (5,0 - 18,0)	673 (100)	8,0 (4,0, 14,0)	673 (100)	<0,001
Mortalidade hospitalar	135 (18,9)	673 (100)	128 (18,0)	673 (100)	0,682
**Características**	**HCQ/CQ + AZT^†^ N = 559^1^**	**Casos válidos n (%)**	**Controles^††^ N = 559^1^**	**Casos válidos n (%)**	**Valor p^2^**
Admissão na UTI	196 (35,1)	559 (100)	179 (32,0)	559 (100)	0,282
Diálise durante internação	55 (9,8)	559 (100)	109 (9,7)	559 (100)	0,920
Ventilação mecânica	145 (27,0)	538 (96,0)	120 (22,3)	539 (96,0)	0,074

* Pareados por idade, sexo, número de comorbidades cardiovasculares, hospital de origem, uso de corticoide. ^1^Mediana (intervalo interquartil); n (%) ^2^teste de Wilcoxon; teste do qui-quadrado de Pearson; teste exato de Fisher; UTI: Unidade de Terapia Intensiva. ^†^Esta amostra exclui pacientes que receberam hidroxicloroquina ou cloroquina com ou sem azitromicina após admissão na unidade de terapia intensiva, ventilação mecânica invasiva e diálise. ^††^Controles pareados com o grupo de pacientes mencionado acima que receberam hidroxicloroquina ou cloroquina com ou sem azitromicina após admissão na unidade de terapia intensiva, ventilação mecânica invasiva e diálise.

## Discussão

No presente estudo, pacientes com covid-19 tratados com CQ ou HCQ com ou sem AZT apresentaram um maior tempo de internação hospitalar e maior frequência de prolongamento do intervalo QT corrigido em comparação a controles pareados. Admissão em UTI, diálise, ventilação mecânica invasiva e mortalidade hospitalar não foram estatisticamente diferentes entre os grupos.

Quanto à prevalência de comorbidades individuais, mais de 60% dos pacientes em tratamento com CQ ou HCQ apresentaram pelo menos uma comorbidade cardiovascular, sendo hipertensão a mais comum. O perfil dos pacientes recebendo CQ e HCQ na presente coorte foi consideravelmente diferente dos pacientes incluídos em alguns dos maiores ECRs sobre o assunto, como o ensaio RECOVERY,^5^ no qual apenas 27% dos pacientes apresentaram comorbidades cardíacas, e o SOLIDARITY,^6^ no qual 21% dos pacientes foram previamente diagnosticados com doença cardíaca. Tal fato possivelmente se explica por viés de seleção, uma vez que as considerações éticas para entrar nos ensaios clínicos excluíram aqueles com risco adicional de apresentar efeitos adversos graves pelo uso da medicação, como pacientes com comorbidades cardíacas.^5,6^ Assim, como esses medicamentos são muito usados fora de ensaios clínicos, a análise dos pacientes em um cenário não controlado é necessária e apropriada para confirmar evidências de ECRs.

Ao receber HCQ ou CQ, principalmente associada à AZT, os pacientes devem ser monitorizados quanto ao desenvolvimento de eventos adversos cardíacos. No contexto da covid-19, protocolos sobre o uso de medicamentos durante a internação recomendam a realização de ECG antes de iniciar a terapia. Isso é ainda mais importante em pacientes com comorbidades cardíacas (60% de nossa amostra), já que o ECG permite a exclusão de anormalidades eletrocardiográficas que possam contraindicar a terapia. Contudo, mesmo com essas recomendações, apenas 42,3% dos pacientes apresentaram ECG à admissão. Entre os que apresentaram registro de ECG, 8,1% dos pacientes no grupo HCG/CQ + AZT não apresentaram ritmo sinusal à admissão, sete pacientes tinham diagnóstico de fibrilação ou *flutter* atrial, e três apresentavam ritmo de marca-passo, o que demonstra que o tratamento foi prescrito mesmo a pacientes com alterações eletrocardiográficas graves.

Quanto aos desfechos eletrocardiográficos durante a internação, prolongamento do intervalo QT foi mais frequente em pacientes usando a medicação em comparação aos controles (3,6 vs. 0,4, p<0,001). Sabe-se que tanto a HCQ como a CQ têm o potencial de prolongar o intervalo QT, e foi demonstrado que a AZT tem um impacto sobre desfechos cardiovasculares e incidência de parada cardíaca. Quando administrados simultaneamente, o potencial para toxicidade cardíaca parece aumentar. Evidência de uma revisão sistemática que incluiu 47 estudos e um total de 13087 pacientes mostrou que pacientes tratados com HCQ e AZT apresentaram maior risco de apresentar prolongamento do intervalo QTc (risco relativo [RR], 3,28; IC95%, 1,16-9,30), o que está de acordo com os nossos achados. Em nosso estudo, não conseguimos identificar diferenças na ocorrência de arritmias malignas entre o grupo de estudo e o grupo controle, o que sugere que o prolongamento do QT pode servir como um preditor precoce de arritmias malignas. Consequentemente, ele pode atuar como marcador na prática clínica, merecendo mais atenção.

Em nosso estudo, mortalidade hospitalar, admissão em UTI, necessidade de diálise ou de ventilação mecânica não foram semelhantes entre os grupos. Evidência prévia corrobora com a ausência de benefício da terapia no tratamento de covid-19, com estudos mostrando até evidência de aumento na mortalidade (prejuízo).^7,15-17^ Uma revisão sistemática de 29 estudos (três ECRs, um ensaio não randomizado e 25 estudos observacionais) com 11932 pacientes mostrou que a terapia com HCQ isolada não foi associada com maior sobrevida [RR combinado, 0,83 (IC 95% 0,65 – 1,06, n=17 estudos)] e um maior risco de morte foi observado nos 8081 pacientes tratados com HQC e AZT.^16^ No entanto, essa revisão foi conduzida antes da publicação do RECOVERY e do SOLIDARITY.^5,6^ Com esse propósito, outra revisão sistemática foi conduzida, incluindo somente ECRs; 28 ECRs (14 publicados e 14 não publicados) com 10319 pacientes foram incluídos, comparando HCQ ou CQ com tratamento padrão ou placebo.^18^ Essa revisão encontrou uma mortalidade aumentada entre os pacientes tratados com HCQ, com um OR para mortalidade por todas as causas de 1,11 (IC95%: 1,02, 1,20; I^2^ = 0%; 26 ensaios; 10,012 pacientes) e nenhum benefício com CQ, com um OR de 1,77 (IC95%: 0,15, 21,13, I^2^ = 0%; quatro ensaios; 307 patients).^18^ No entanto, o estudo não incluiu o efeito da associação da AZT com HCQ/CQ sobre a mortalidade.

Um ECR foi conduzido com 504 pacientes brasileiros com covid-19, alocados em uma relação 1:1:1 para receberem tratamento padrão, tratamento padrão mais HCQ, ou tratamento padrão mais HCQ e AZT. Os pacientes que receberam HCQ com ou sem AZT apresentaram maior frequência de prolongamento do intervalo QT e a terapia não pareceu estar associada com melhora do estado do paciente durante a internação.^19^ Outro estudo brasileiro não randomizado, com pacientes com covid-19 em tratamento ambulatorial mostrou que a CQ foi independentemente associada com mortalidade mais alta (OR 1,67 [IC 95% 1,20-2,28]), mas não foi associada com ocorrência de alterações eletrocardiográficas importantes (OR = 0,80 [IC95% 0,63-1,02)].^20^Uma análise retrospectiva de pacientes internados com covid-19 que usaram HCQ também detectou uma taxa mais alta de mortalidade (OR 3,3; IC95% 1,1-9,6; p=0,03).^21^

A maioria da evidência robusta existente sobre HCQ/CQ e ventilação mecânica invasiva deriva do ensaio RECOVERY,^5^ o maior ECR até o momento, que, sozinho, representou 47% da amostra e 76% do peso da revisão sistemática mencionada acima que incluiu 28 ECRs.^18^ Nesse estudo, que incluiu 1561 pacientes tratados com HCQ, comparados a 3155 pacientes que receberam tratamento usual, o grupo que recebeu HCQ apresentou uma frequência mais alta de desfecho composto de ventilação mecânica invasiva ou morte (30,7% vs. 26,9%, RR = 1,14, IC95% 1,03 – 1,27).^5^ Contudo, esse estudo se difere do nosso, uma vez que considera o uso de HCQ ou de CQ isolada em comparação a tratamento padrão, ao passo que, em nossa amostra, o esquema terapêutico mais popular (recebido por 90,9% dos pacientes na amostra) incluiu a associação com AZT. Nossa hipótese é que o efeito sobre a ventilação mecânica (HCQ/CQ 27,0% vs. controles 22,3%, p=0,074) na presente análise pode não ter sido percebido devido ao tamanho limitado da amostra (n = 559 pacientes). A possibilidade de uma maior frequência de ventilação mecânica invasiva é muito relevante, não só pelo maior risco de complicações imediatas, como infecção nosocomial, como também pelo consumo de recursos e risco de piores desfechos a longo prazo. Um estudo multicêntrico brasileiro recente acompanhou 1508 pacientes com covid-19 por até um ano de internação e observou que aqueles que necessitaram de ventilação mecânica durante a internação apresentaram escores de utilidade relacionados à qualidade de vida mais baixos, mortalidade por todas as causas mais alta (7,9% vs. 1,2%; diferença ajustada, 7,1% [IC95% 2,5%–11,8%]), maior frequência de eventos cardiovasculares (5,6% vs. 2,3%; diferença ajustada, 2,6% [IC95% 0,6%–4,6%]) e novas incapacidades em atividades instrumentais diárias (40,4% vs. 23,5%; diferença ajustada, 15,5% [IC95% 8,5%–22,.5]) em um ano de seguimento.^22^

Este estudo apresentou uma visão abrangente do uso da HCQ ou CQ em uma grande coorte de hospitais brasileiros. Apesar de seus pontos fortes, tais como tamanho amostral, análise pareada e delineamento multicêntrico, o presente estudo tem limitações que precisam ser abordadas. Uma vez que inclui dados retrospectivos coletados de prontuários médicos, os resultados estão sujeitos a algumas desvantagens inerentes aos dados. Para minimizar tal fato, todos os profissionais de saúde e alunos de graduação responsáveis por reunir os dado participaram de treinamento mandatório em como coletar dados de prontuários médicos. Ainda, uma vez que se trata de estudo observacional não randomizado, não foi possível estabelecer associações de causa e efeito. Além disso, os dados do estudo refletem um período anterior à vacinação na população brasileira e antes que terapias antivirais eficazes estivessem disponíveis. Outros tratamentos usados durante a internação hospitalar, incluindo anticoagulação, esteroides e anticorpos monoclonais não eram padronizados. Embora os resultados possam não refletir o perfil atual e o prognóstico dos pacientes com covid-19, as limitações não invalidam os resultados do estudo quanto à HCQ/CQ. Finalmente, a ocorrência de viés pode ser uma limitação. Por se tratar de um contexto de uso compassivo, o medicamento pode ter sido oferecido a indivíduos altamente selecionados, tais como pacientes com condições mais graves e menos propensos a apresentarem efeitos adversos. Contudo, é provável que o pareamento bem-sucedido entre pacientes usando HCQ/CQ e controles tenham diminuído possíveis riscos de viés de indicação e que o viés não tenha interferido ou invalidado os resultados do estudo.

## Conclusão

Neste estudo, os pacientes tratados com CQ ou HCQ com ou sem AZT apresentaram maior tempo de internação hospitalar, em comparação aos controles pareados. As alterações eletrocardiográficas foram mais prevalentes nos pacientes com covid-19 usando CQ em comparação a controles. Entretanto, não foram observadas diferenças quanto à ventilação mecânica, admissão em UTI, diálise e mortalidade hospitalar entre os dois grupos. Este estudo corrobora o volume de corpo de evidências existentes, que não apoiam o uso desses medicamentos no tratamento de pacientes com covid-19.
