# Supplementary material for: Dados de Vida Real sobre o Uso da Hidroxicloroquina ou da Cloroquina Combinadas ou Não à Azitromicina em Pacientes com Covid-19: Uma Análise Retrospectiva no Brasil
Source: Arq Bras Cardiol. 2023 Oct 3;120(9):e20220935. [Article in Portuguese] doi: 10.36660/abc.20220935 (PMC10547436; doi:10.36660/abc.20220935)
Supplement: Translation

# Translation: Real-Life Data on Hydroxychloroquine or Chloroquine with or Without Azithromycin in COVID-19 Patients: A Retrospective Analysis in Brazil

**Authors:** Maíra Viana Rego Souza-Silva, Daniella Nunes Pereira, Magda Carvalho Pires, Isabela Muzzi Vasconcelos, Alexandre Vargas Schwarzbold, Diego Henrique de Vasconcelos, Elayne Crestani Pereira, Euler Roberto Fernandes Manenti, Felício Roberto Costa, Filipe Carrilho de Aguiar, Fernando Anschau, Frederico Bartolazzi, Guilherme Fagundes Nascimento, Heloisa Reniers Vianna, Joanna d’Arc Lyra Batista, Juliana Machado-Rugolo, Karen Brasil Ruschel, Maria Angélica Pires Ferreira, Leonardo Seixas de Oliveira, Luanna Silva Monteiro Menezes, Patricia Klarmann Ziegelmann, Marcela Gonçalves Trindade Tofani, Maria Aparecida Camargos Bicalho, Matheus Carvalho Alves Nogueira, Milton Henriques Guimarães-Júnior, Rúbia Laura Oliveira Aguiar, Danyelle Romana Alves Rios, Carisi Anne Polanczyk, Milena Soriano Marcolino

**Affiliations:** 1 Brasil Faculdade de Medicina – Universidade Federal de Minas Gerais, Belo Horizonte, MG – Brasil; 2 Brasil Departamento de Estatística – Instituto de Ciências Exatas (ICEx) – Universidade Federal de Minas Gerais, Belo Horizonte, MG – Brasil; 3 Brasil Hospital Universitário de Santa Maria (EBSERH) – Universidade Federal de Santa Maria, Santa Maria, RS – Brasil; 4 Brasil Hospital SOS Cárdio, Florianópolis, SC – Brasil; 5 Brasil Instituto de Medicina Vascular – Hospital Mãe de Deus, Porto Alegre, RS – Brasil; 6 Brasil Hospital Municipal Odilon Behrens, Belo Horizonte, MG – Brasil; 7 Brasil Hospital das Clínicas da Universidade Federal de Pernambuco, Recife, PE – Brasil; 8 Brasil Programa de Avaliação e Produção de Tecnologias para o Sistema Único de Saúde - Hospital Nossa Senhora da Conceição e Hospital Cristo Redentor, Porto Alegre, RS – Brasil; 9 Brasil Hospital Santo Antônio, Curvelo, MG – Brasil; 10 Brasil Hospital Unimed, Belo Horizonte, MG – Brasil; 11 Brasil Hospital Universitário Ciências Médicas, Belo Horizonte, MG – Brasil; 12 Brasil Hospital Mãe de Deus – Universidade Federal da Fronteira Sul, Chapecó, SC – Brasil; 13 Brasil Instituto de Avaliação de Tecnologia em Saúde (IATS/CNPq), Porto Alegre, RS – Brasil; 14 Brasil Hospital das Clínicas da Faculdade de Medicina de Botucatu, Botucatu, SP – Brasil; 15 Brasil Hospital Mãe de Deus – Hospital Universitário de Canoas - Universidade Federal do Rio Grande do Sul, Porto Alegre, RS – Brasil; 16 Brasil Instituto de Avaliação de Tecnologia em Saúde (IATS/CNPQ), Porto Alegre, RS – Brasil; 17 Brasil Hospital de Clínicas de Porto Alegre, Porto Alegre, RS – Brasil; 18 Brasil Hospital Santa Rosália, Teófilo Otoni, MG – Brasil; 19 Brasil Hospital Luxemburgo, Belo Horizonte, MG – Brasil; 20 Brasil Hospital Tacchini, Bento Gonçalves, RS – Brasil; 21 Brasil Hospital Semper, Belo Horizonte, MG – Brasil; 22 Brasil Departamento de Medicina Interna, Faculdade de Medicina - Hospitais Fundação Hospitalar do Estado de Minas Gerais (FHEMIG), Belo Horizonte, MG – Brasil; 23 Brasil Hospitais da Rede Mater Dei, Belo Horizonte, MG – Brasil; 24 Brasil Hospital Márcio Cunha, Ipatinga, MG – Brasil; 25 Brasil Hospital Metropolitano Doutor Célio de Castro, Belo Horizonte, MG – Brasil; 26 Brasil Universidade Federal de São João del-Rei, Divinópolis, MG – Brasil; 27 Brasil Universidade Federal do Rio Grande do Sul, Porto Alegre, RS – Brasil; 28 Brasil Instituto de Avaliação de Tecnologias em Saúde (IATS/CNPq), Porto Alegre, RS – Brasil; 29 Brasil Hospital Moinhos de Vento, Porto Alegre, RS – Brasil; 30 Brasil Departamento de Clínica Médica – Faculdade de Medicina – Universidade Federal de Minas Gerais, Belo Horizonte, MG – Brasil; 31 Brasil Centro de Telessaúde do Hospital Universitário da Universidade Federal de Minas Gerais, Belo Horizonte, MG – Brasil

**Keywords:** Chloroquine, Hydroxychloroquine, Azithromycin, COVID-19

## Abstract

**Background:**

Despite no evidence showing benefits of hydroxychloroquine and chloroquine with or without azithromycin for COVID-19 treatment, these medications have been largely prescribed in Brazil.

**Objectives:**

To assess outcomes, including in-hospital mortality, electrocardiographic abnormalities, hospital length-of-stay, admission to the intensive care unit, and need for dialysis and mechanical ventilation, in hospitalized COVID-19 patients who received chloroquine or hydroxychloroquine, and to compare outcomes between those patients and their matched controls.

**Methods:**

A retrospective multicenter cohort study that included consecutive laboratory-confirmed COVID-19 patients from 37 Brazilian hospitals from March to September 2020. Propensity score was used to select matching controls by age, sex, cardiovascular comorbidities, and in-hospital use of corticosteroid. A p-value <0.05 was considered statistically significant.

**Results:**

From 7,850 COVID-19 patients, 673 (8.6%) received hydroxychloroquine and 67 (0.9%) chloroquine. The median age in the study group was 60 years (46 - 71) and 59.1% were women. During hospitalization, 3.2% of patients presented side effects and 2.2% required therapy discontinuation. Electrocardiographic abnormalities were more prevalent in the chloroquine/hydroxychloroquine group (13.2% vs. 8.2%, p=0.01), and the long corrected QT interval was the main difference (3.6% vs. 0.4%, p<0.001). The median hospital length of stay was longer in the HCQ/CQ + AZT group than in controls (9.0 [5.0, 18.0] vs. 8.0 [4.0, 14.0] days). There was no statistical differences between groups in intensive care unit admission (35.1% vs. 32.0%; p=0.282), invasive mechanical ventilation support (27.0% vs. 22.3%; p=0.074) or mortality (18.9% vs. 18.0%; p=0.682).

**Conclusion:**

COVID-19 patients treated with chloroquine or hydroxychloroquine had a longer hospital length of stay, when compared to matched controls. Intensive care unit admission, invasive mechanical ventilation, dialysis and in-hospital mortality were similar.

## Introduction

The COVID-19 pandemic caused an unprecedented global effort in the search for effective treatments to fight the disease. In this context, hydroxychloroquine (HCQ) and chloroquine (CQ) have caught the attention of the scientific community due to *in vitro* evidence showing an antiviral activity and immunomodulatory effect, which, in theory, could prevent cytokine storm.^1^ *In vitro* studies also showed that azithromycin (AZT) could have a synergic effect on the HCQ/CQ effects against SARS-CoV-2.^2^ On top of that, both medications are affordable, have a well-known safety profile, and are readily available all over the world, so they soon became potential treatments to the disease.^2^

Considering this encouraging preliminary information, important regulatory agencies, such as the Food and Drug Administration (FDA) and the European Medicines Agency (EMA), granted permission for the use of HCQ and CQ outside clinical trials in March 2020, due to the emergency situation.^3,4^

Currently, even with robust findings from large randomized clinical trials (RCT), such as RECOVERY^5^ and SOLIDARITY-WHO,^6^ and several meta-analyses, showing no evidence to support this therapy and even evidence of harm,^7,8^ some physicians and Brazilian guidelines kept recommending the use of this medication for COVID-19 treatment.^9^ Therefore, this study aims to assess the clinical and electrocardiographic outcomes of hospitalized COVID-19 patients, who received CQ or HCQ, with or without AZT, from a large cohort of Brazilian hospitals, as well as to compare outcomes between those patients and their matched controls.

## Methods

### Study design

The present study is a part of the Brazilian COVID-19 Registry, a retrospective multicenter cohort study, which enrolled consecutive patients with laboratory-confirmed COVID-19.^10,11^ This study adheres to the STROBE guidelines (*Strengthening the Reporting of Observational Studies in Epidemiology*).^12^ The Brazilian National Commission for Research Ethics (CAAE 30350820.5.1001.0008) approved the development of this study and the need for individual consent was waived due to the pandemic circumstances and analysis based only on unidentified patient data.

The present analysis included patients admitted to 37 participant hospitals in 17 Brazilian cities, from March to September, 2020.^10,11^ There were no losses due to the retrospective nature of the study. Patients with missing values in the different variables were not excluded, and there were no missing values related to the exposure. We chose not to apply any further exclusion criteria, as our aim is to provide a real-life observational report.

### Data collection

Trained researchers collected patient data from the medical records using the Research Data Capture (REDCap®) electronic data capture system,^13,14^ hosted at the Telehealth Center of the University Hospital of the *Universidade Federal de Minas Gerais*.^14^ Data included patients’ demographic and clinical characteristics, clinical evaluation at hospital presentation, laboratory, imaging, and electrocardiographic data, therapeutic interventions, and outcomes.^10^ To ensure reliability and monitor data quality, all information underwent an automatic verification periodically, to identify outliers and possible inconsistencies.

### Outcomes

The primary outcome was COVID-19 in-hospital mortality. Secondary outcomes included electrocardiographic abnormalities (rhythm, heart rate, corrected QT interval, structural abnormalities, blocks, and tachycardias), hospital length-of-stay, admission to the intensive care unit (ICU), and need for dialysis and for invasive mechanical ventilation.

### Statistical analysis

For the descriptive analysis, the demographic and clinical characteristics of the patients were represented by frequency distribution, using median and interquartile range for continuous variables, as they did not present normal distribution, and numbers and percentages for counts. Patients who received HCQ or CQ with or without AZT (HCQ/CQ + AZT) at any dosage were compared to matched controls (patients who did not receive this treatment) using the chi-square test and the Fisher’s exact test for categorical variables and the Wilcoxon rank sum test for continuous variables. In the latter case, the Kolmogorov-Smirnov test was applied to verify data normality.

For comparison purposes, a propensity score model (including age, sex, the number of cardiovascular comorbidities, hospital of origin, and corticoid use) was estimated by logistic regression to adjust potential confounding variables and match patients to controls. The control group was those who had the closest propensity score to those treated with CQ (within 0.17 standard deviations of the logit of the propensity score, on a scale from 0-1.00), using the MatchIt package, in R software.

For the outcomes admission to the ICU, and need for dialysis and mechanical ventilation, patients who used HCQ/CQ + AZT after the occurrence of these outcomes were excluded.

Results were considered statistically significant at a significance level of 5%. Statistical analysis was performed with the R program for statistical computing (version 4.0.2).

## Results

From 7,850 hospitalized patients with a confirmed COVID-19 diagnosis, 725 (9.2%) patients received CQ/HCQ with or without AZT for COVID-19 treatment during hospital stay, 659 (90.9%) were treated with HCQ, 67 (9.2%) with CQ, and 640 (88.3%) received AZT simultaneously (Supplementary table 1). Of those, 673 patients who received CQ/HCQ could be matched, and the accuracy of the final propensity model was 0.91. The standardized mean differences of the key covariates before and after matching are shown in Supplementary table 2 and on the Central Figure.

The prevalence of comorbidities was similar between the two matched groups, as shown in Table 1, except for chronic kidney disease, which was more common in the controls. As for clinical characteristics at admission, the HCQ/CQ + AZT group had a slightly decreased SpO_2_/FiO_2_ ratio compared to controls. Characteristics of patients who were assessed for invasive mechanical ventilation, dialysis and ICU admission are described in Supplementary table 3.

**Table 1:** – Demographic and clinical characteristics of COVID-19 patients on hydroxychloroquine (HCQ)/chloroquine (CQ) therapy and matched controls*

Characteristics	HCQ/CQ + with our without AZT N= 673^1^	Controls N= 673^1^	p-value^2^
**Age (years)**	58.0 (46. 70)	60.0 (46. 71)	0.186
**Female sex**	376 (55.9)	398 (59.1)	0.247
**Cardiovascular diseases**			
Hypertension	335 (49.8)	351 (52.2)	0.413
Chronic heart failure	37 (5.5)	33 (4.9)	0.713
Coronary artery disease	26 (3.9)	23 (3.4)	0.771
Ischemic stroke	18 (2.7)	20 (3.0)	0.869
Atrial fibrillation or flutter	17 (2.5)	14 (2.1)	0.716
**Respiratory diseases**			
Asthma	53 (7.9)	38 (5.6)	0.129
COPD	45 (6.7)	33 (4.9)	0.199
**Metabolic diseases**			
Diabetes mellitus	196 (29.1)	179 (26.6)	0.331
Obesity	133 (19.8)	113 (16.8)	0.180
**Other conditions**			
Psychiatric disease	48 (7.1)	44 (6.5)	0.746
Active cancer	40 (5.9)	57 (8.5)	0.911
Chronic kidney disease	30 (4.5)	49 (7.3)	0.037
Rheumatological disease	21 (3.1)	14 (2.1)	0.304
Cirrhosis	3 (0.4)	5 (0.7)	0.726
**Clinical characteristics at presentation**			
Invasive mechanical ventilation	52 (7.3)	46 (6.5)	0.591
SpO_2_/FiO_2_	438.1 (317.0. 457.1)	447.6 (335.7. 461.9)	0.011
**Lifestyle habits**			
Alcohol use disorder	15 (2.2)	25 (3.7)	0.149
Smoking	85 (12.6)	87 (12.9)	0.935
**Medication during hospital stay**			
Antibiotics (except azithromycin)	550 (81.7)	522 (77.6)	
Anticoagulants	596 (88.6)	578 (85.9)	
Corticosteroids	375 (55.7)	356 (52.9)	

* Matched by age, sex, the number of cardiovascular comorbidities, hospital of origin, and corticoid use. ^1^n (%); Median (IQR) ^2^Pearson’s Chi-squared test; Wilcoxon rank sum test; Fisher’s exact test; AZT: azithromycin; COPD: chronic obstructive pulmonary diseases; SpO_2_: peripheral oxygen saturation; FiO_2_: fraction of inspired oxygen.

With regards to the posology (Table 2), most patients used HCQ (90.1%) with or without AZT at a dose of 850 mg on the first day, followed by 450 mg daily for the duration of the treatment. The median length of the treatment was five days. Thirty patients presented side effects that were attributed to the medication by the attending medical team, with QT interval prolongation and gastrointestinal symptoms as the most common ones. The therapy was suspended in 15 patients due to concerns with side effects.

**Table 2:** – Chloroquine and hydroxychloroquine posology and side effects in COVID-19 patients (n=673)

Medication used	n (%)
Chloroquine	65 (9.9)
Hydroxychloroquine	612 (90.1)
With azithromycin	598 (88.1)
**Chloroquine’s posology**	
450 mg every 12 hours on day 1 + 450 mg every 24 hours on the following days	45 (67.2)
500 mg every 12 hours on day 1 + 250 mg every 12 hours on the following days	7 (10.4)
Other	15 (23.4)
Duration of the therapy (days)	5.0 (3.0 - 6.0)
**Hydroxychloroquine’s posology**	
400 mg every 12 hours on day 1 + 200 mg every 12 hours on the following days	252 (59.6)
400 mg every 24 hours	149 (35.2)
400 mg every 12 hours on day 1 + 200 mg every 8 hours on the following days	21 (5.0)
200 mg every 8 hours	1 (0.2)
Other	195 (46.1)
Duration of the therapy (days)	5.0 (4.0 - 6.0)
Medication side effects	30 (4.4)
Monomorphic ventricular tachycardia	1 (0.1)
QT interval prolongation	16 (2.4)
Gastrointestinal symptoms	5 (0.7)
Atrial fibrillation/flutter	2 (0.3)
Non-ischemic cardiac dysfunction	1 (0.1)
Other side effects	8 (1.2)
Therapy suspension due to side effects	15 (2.2)

Less than half of patients on therapy had an electrocardiogram (ECG) recorded in the first 24 hours after admission. Most patients were on sinus rhythm and primary repolarization abnormality was the most common electrocardiographic finding.

For ECG outcomes (Table 3), patients in the study group were more likely to be diagnosed with a novel electrocardiographic abnormality during hospital stay. QT interval prolongation was the most frequent abnormality and its frequency was higher in patients on HCQ/CQ compared to controls. Supraventricular tachycardia was more frequent in controls. There were no other statistically significant differences in electrocardiographic abnormalities between the groups.

**Table 3:** – Electrocardiographic findings upon admission and outcomes during hospital stay of COVID-19 patients treated with hydroxychloroquine (HCQ) / chloroquine (CQ) with or without azythromicyn (AZT) and controls

Characteristics	HCQ/CQ + with our without AZT N = 673^1^	Controls N = 673^1^	p-value^2^
Electrocardiogram at admission	285 (42.3)	182 (27.0)	<0.001
Cardiac rate, bpm	83.0 (74.0. 91.0)	85.0 (74.0. 96.0)	0.063
QT interval, ms	360.0 (320.0. 406.0)	360.0 (320.0. 400.0)	0.060
Sinus rhythm	262 (91.9)	155 (85.2)	0.016
Primary repolarization abnormalities	78 (27.6)	50 (27.5)	>0.999
Right bundle branch block	13 (4.6)	13 (7.1)	0.337
Atrial fibrillation/flutter	7 (2.5)	9 (4.9)	0.249
Left anterior hemiblock	6 (2.1)	6 (3.3)	0.630
Left bundle branch block	4 (1.4)	3 (1.6)	>0.999
Left ventricular hypertrophy with ST-T abnormalities	4 (1.4)	3 (1.6)	>0.999
Pacemaker rhythm	3 (1.1)	3 (1.6)	0.683
First-degree atrioventricular block	2 (0.7)	3 (1.6)	0.384
Pathological Q waves	1 (0.4)	0 (0.0)	>0.999
Monomorphic ventricular tachycardia	0 (0.0)	1 (0.5)	0.153
Supraventricular tachycardia	0 (0.0)	1 (0.5)	0.153
Total atrioventricular block	0 (0.0)	2 (1.1)	0.153
Electrocardiographic outcomes	89 (13.2)	55 (8.2)	0.004
Atrial fibrillation/flutter	24 (3.6)	20 (3.0)	0.646
QT interval prolongation	26 (3.6)	3 (0.4)	<0.001
Primary repolarization abnormalities	16 (2.4)	12 (1.8)	0.567
Left ventricular hypertrophy with ST-T abnormalities	5 (0.7)	5 (0.7)	>0.999
First-degree AV block	5 (0.7)	4 (0.6)	>0.999
Right bundle branch block	5 (0.7)	2 (0.3)	0.452
Ventricular extrasystoles	2 (0.3)	5 (0.7)	0.452
Pacemaker rhythm	2 (0.3)	2 (0.3)	>0.999
Polymorphic ventricular tachycardia	2 (0.3)	2 (0.3)	>0.999
Left bundle branch block	2 (0.3)	2 (0.3)	>0.999
Left anterior hemiblock	1 (0.1)	1 (0.1)	>0.999
Supraventricular extrasystoles	1 (0.1)	1 (0.1)	>0.999
Second-degree atrioventricular block	1 (0.1)	0 (0.0)	>0.999
Supraventricular tachycardia	0 (0.0)	6 (0.9)	0.031

Matched by age, sex, the number of cardiovascular comorbidities, hospital of origin, and corticoid use. ^1^n (%); Median (IQR) ^2^Pearson’s Chi-squared test; Wilcoxon rank sum test; Fisher’s exact test

With regards to the outcomes (Table 4), the median hospital length of stay was longer in the HCQ/CQ + AZT group than in controls. There were no statistically significant differences in the need for admission to an ICU, mechanical ventilation or dialysis, and in-hospital mortality between groups.

**Table 4:** – Comparison of clinical outcomes of COVID-19 patients on hydroxychloroquine (HCQ) / chloroquine (CQ) with or without azythromicyn (AZT) and controls*

Characteristics	HCQ/CQ + with or without AZT N = 673^1^	Non-missing cases n (%)	Controls N = 673^1^	Non-missing cases n (%)	p-value^2^
Tempo de internação (dias)	9.0 (5.0 - 18.0)	673 (100)	8.0 (4.0. 14.0)	673 (100)	<0.001
Mortalidade hospitalar	135 (18.9)	673 (100)	128 (18.0)	673 (100)	0.682
**Characteristics**	**HCQ/CQ + AZT^†^ N = 559^1^**	**Non-missing cases n (%)**	**Controls^††^ N = 559^1^**	**Non-missing cases n (%)**	**p-value^2^**
ICU admission	196 (35.1)	559 (100)	179 (32.0)	559 (100)	0.282
In-hospital dialysis	55 (9.8)	559 (100)	109 (9.7)	559 (100)	0.920
Mechanical ventilation	145 (27.0)	538 (96.0)	120 (22.3)	539 (96.0)	0.074

Matched by age, sex, the number of cardiovascular comorbidities, hospital of origin, and corticoid use. ^1^Median (IQR); n (%) ^2^Wilcoxon rank sum test; Pearson’s Chi-squared test; Fisher’s exact test; ICU: intensive care unit. ^†^This sample excludes patients who received hydroxychloroquine or chloroquine with or without azithromycin after intensive care unit admission, invasive mechanical ventilation and dialysis. ^††^Controls matched to the aforementioned group of patients who received hydroxychloroquine or chloroquine with or without azithromycin after intensive care unit admission, invasive mechanical ventilation and dialysis.

## Discussion

In the present study, COVID-19 patients treated with CQ or HCQ with or without AZT had a longer hospital stay and a higher frequency of long corrected QT interval compared to matched controls. Admission in ICU, dialysis, invasive mechanical ventilation support and in-hospital mortality were not statistically different between the groups.

As for the prevalence of underlying individual comorbidities, it is worth noting that more than 60% of patients on CQ or HCQ had at least one cardiovascular comorbidity, with hypertension as the most common one. The profile of patients receiving CQ and HCQ in our cohort differed considerably from patients enrolled in some of the largest RCTs on the topic, such as the RECOVERY^5^ trial, where only 27% of patients had cardiac comorbidities, and SOLIDARITY,^6^ in which 21% of patients were previously diagnosed with cardiac disease. This is possibly a selection bias as the ethical considerations for entering clinical trials excluded those patients with an additional risk of having serious adverse effects with the medication, such as those with cardiac comorbidities.^5,6^Thus, as these medications were largely used outside clinical trials, the analysis of real-life patients is warranted and appropriate to confirm the existing evidence from RCTs.

When receiving HCQ or CQ, especially associated with AZT, patients should be monitored for the development of cardiac adverse events. In the context of the COVID-19 in-hospital use of these medications, protocols advise that it is recommended to have an ECG recorded before the beginning of the therapy. This is even more important in patients with cardiac comorbidities (60% of our sample), for whom an ECG will exclude electrocardiographic abnormalities that may be a contraindication to therapy. However, even with such recommendations, only 42.3% of patients had an ECG recorded at admission. Among those who had an ECG recorded, 8.1% of patients in the HCQ/CQ + AZT group did not have a sinus rhythm on admission, seven patients had a diagnosis of atrial fibrillation or flutter and three had a pacemaker rhythm, which shows that even patients with serious ECG abnormalities were prescribed the treatment.

With regards to electrocardiographic outcomes during the hospital stay, QT interval prolongation was more common in patients using the therapy compared to controls (3.6 vs. 0.4, p<0.001). It is known that both HCQ and CQ have the potential of prolonging the QT interval and AZT has also been shown to impact cardiovascular outcomes and the incidence of sudden cardiac arrest. When administered simultaneously, the potential for cardiac toxicity seems to increase. Evidence from a systematic review that included 47 studies with a total of 13,087 patients showed that patients who had therapy with HCQ plus AZT had greater risk to be detected with QTc prolongation (relative risk [RR], 3.28; 95% CI, 1.16–9.30), which is in accordance with our findings. In our study, we were unable to identify differences in the occurrence of malignant arrhythmias between the study and control groups, which suggests that QT prolongation may serve as an early predictor of malignant arrhythmias. Consequently, it may be valuable as a surrogate marker in clinical practice, warranting further attention.

In our study, in-hospital mortality, ICU admission, need for mechanical ventilation and dialysis were not different between groups. Previous evidence agrees on the lack of benefit of the therapy for treating COVID-19, with studies showing even evidence of increased mortality (harm).^7,15-17^ A systematic review of 29 studies (three RCTs, one non-randomized trial, and 25 observational studies), with 11,932 patients found that the therapy with HCQ alone was not associated with improved survival (pooled RR, 0.83 [95% CI 0.65-1.06, n= 17 studies]), while among the 8,081 patients who had HCQ with AZT, a higher risk of death (RR= 1.27; 95% CI 1.04-1.54, n= 7 studies) was identified.^16^ However, this review was conducted before the publication of RECOVERY and SOLIDARITY.^5,6^ For that purpose, another systematic review was conducted including only RCTs; 28 studies (14 published and 14 unpublished RCTs) with 10,319 patients were included, comparing HCQ or CQ with standard care or placebo.^18^ This review found an increased mortality among patients receiving HCQ, with an pooled OR for all-cause mortality of 1.11 (95% CI: 1.02, 1.20; I^2^ = 0%; 26 trials; 10,012 patients), and no benefit from CQ, with a pooled OR of 1.77 (95% CI: 0.15, 21.13, I^2^ = 0%; four trials; 307 patients).^18^ However, this study did not evaluate the effect of the combination of AZT with HCQ/CQ on mortality.

A RCT was conducted in 504 Brazilian COVID-19 patients, who were assigned in a 1:1:1 ratio to receive standard care, standard care plus HCQ, or standard care plus HCQ and AZT. Those using HCQ with or without AZT showed a higher frequency of QT interval prolongation, and the therapy did not seem to be associated with an improvement of the patient’s status during hospitalization.^19^ Another Brazilian study, with non-randomized design, on COVID-19 outpatients showed that CQ was independently associated with higher mortality (OR 1.67 [95% CI 1.20-2.28]), but it was not associated with the occurrence of major electrocardiographic abnormalities (OR = 0.80 [95% CI 0.63-1.02)].^20^A retrospective analysis of COVID-19 inpatients who used HCQ also detected a higher rate of mortality (OR 3.3, 95%CI 1.1-9.6, p=0.03).^21^

Most of the robust evidence we have on HCQ/CQ and invasive mechanical ventilation comes from the RECOVERY trial,^5^ the largest published RCT to date, that alone represented 47% of the sample and 76% of the weight of the aforementioned systematic review that included 28 RCTs.^18^ In this study, that included 1,561 patients receiving HCQ compared to 3,155 patients receiving usual care, the HCQ group presented a higher frequency of the composite outcome of invasive mechanical ventilation or death (30.7% vs. 26.9%, RR = 1.14, 95% CI 1.03 - 1.27).^5^ However, this study differs from ours because it considers HCQ or CQ alone compared with standard care, while in our sample the most popular treatment scheme (received by 90.9% of patients in the sample) included the association with AZT. We hypothesize that the effect on mechanical ventilation (HCQ/CQ 27.0% vs. controls 22.3%, p=0.074) in the present analysis might not have been perceived due to the limited sample size (n = 559 patients). The possibility of a higher frequency of invasive mechanical ventilation is very relevant, not only due to a higher risk of immediate complications, such as nosocomial infection, but also due to resource depletion and risk of worse long-term outcomes. A recent multicenter Brazilian study followed 1,508 COVID-19 patients up to one year of hospitalization, and observed that those who needed mechanical ventilation during hospitalization had lower health-related quality-of-life utility scores, higher all-cause mortality (7.9% vs 1.2%; adjusted difference, 7.1% [95% CI 2.5%–11.8%]), major cardiovascular events (5.6% vs 2.3%; adjusted difference, 2.6% [95% CI 0.6%–4.6%]), and new disabilities in instrumental activities of daily living (40.4% vs 23.5%; adjusted difference, 15.5% [95% CI 8.5%–22.5]) at one year follow-up.^22^

This study provided a comprehensive overview of CQ or HCQ use in a large cohort of Brazilian hospitals. Even with its strengths, such as sample size, matched analysis, and multicenter design, the present study has limitations that should be addressed. As it includes retrospective data collected from medical records, the results are subjected to drawbacks inherent to the data. To minimize this, extensive training on how to collect data from medical records was mandatory for all health professionals and undergraduate students responsible for data gathering. Furthermore, as this is an observational and non-randomized study, it was not possible to establish cause-effect associations. Also, the study’s data reflect a time period before vaccination was offered to the Brazilian population and before effective antiviral therapies were available. Other treatments used during hospitalization, such as anticoagulation, steroids and monoclonal antibodies were not standardized. Although the results may not reflect the current profile and prognosis of in-hospital COVID-19 patients, the limitations do not invalidate the study’s results with regards to HCQ/CQ. At last, indication bias could be a limitation. As it was a compassionate use context, the drug could be given to highly selected individuals, such as patients who had a more severe condition and less prone to have adverse effects. However, it is likely that the successful matching between patients using HCQ/CQ and controls have lessened possible risks of indication bias, that the bias have not interfered or invalidated the study results.

## Conclusion

In this study, patients treated with CQ or HCQ with or without AZT had a longer length of hospital stay, compared to matched controls. Electrocardiographic abnormalities were more prevalent in COVID-19 patients using CQ than in their controls. Nonetheless, no difference was observed in mechanical ventilation, ICU admission, dialysis and in-hospital mortality between the two groups. The study adds to the bulk of evidence not supporting the use of these medications for COVID-19 patients.
